# Nested TMAPs to Visualize Billions of Molecules

**DOI:** 10.1021/acs.jcim.6c00420

**Published:** 2026-05-04

**Authors:** Alejandro Flores Sepúlveda, Jean-Louis Reymond

**Affiliations:** Department of Chemistry, Biochemistry and Pharmaceutical Sciences, 27210 University of Bern, Freiestrasse 3, 3012 Bern, Switzerland

## Abstract

Here, we present a visualization and clustering framework enabling the exploration of billion-sized chemical data sets, exemplified with the REAL database of 9.6 billion make-on-demand molecules. We represent molecules as 42-dimensional MQN (molecular quantum numbers) fingerprints describing molecular structures with counts for different atom and bond types, polar groups and topological features, and cluster the data set by applying Product Quantization and PQk-Means. We retrieve the molecule closest to the cluster centroid as a representative for each cluster and compute a tree-map (TMAP) displaying these representatives organized by MQN-similarity. Each cluster representative in this primary TMAP is linked to a nested secondary TMAP displaying the corresponding cluster content organized by the ECFP4 substructure fingerprint similarity. This nested TMAP approach can be computed on a single workstation and gives direct access to the entire data set down to single molecular structures in two clicks. A nested TMAP for the REAL database is accessible at https://chelombus.gdb.tools/databases/real-database.

## Graphical abstract

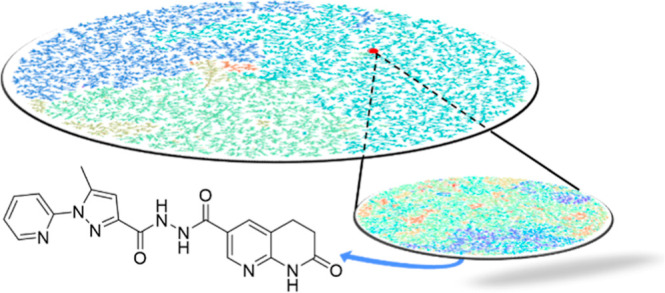

## Introduction

Advances in cheminformatics and data science are transforming chemical space from a theoretical concept into actual and increasingly large molecular data sets, typically billion-sized libraries of screening compounds to support drug discovery.^1–4^ Unfortunately, these data sets are difficult to analyze and understand because clustering algorithms and visualization tools are typically limited to at most a few million molecules. This limitation applies in particular to dimensionality reduction methods for high-dimensional molecular fingerprint representations,^5^ such as principal component analysis (PCA)^6^ and the related similarity maps,^7–10^ self-organizing maps,^11^ generative topographic mapping,^12^ *t*-distributed stochastic neighbor embedding,^13^ uniform manifold approximation and projection (UMAP),^14^ tree-maps (TMAP),^15^ and other tools.^16–21^

In our previously reported TMAP visualization tool, we computed an approximate nearest–neighbor graph of a data set using locality sensitive hashing on vector representations of data set objects, reduced it to the corresponding minimum spanning tree, and computed a 2D-layout of this minimum spanning tree where each node represents a different object, typically a molecule, and each edge represents an approximate nearest neighbor connection.^15^ The TMAP was displayed in an interactive format in a web browser using the applications Faerun and SmilesDrawer, in which each molecule can be viewed individually.^22,23^ Unfortunately, the TMAP code only runs well for data sets of up to a few million molecules, and the display is only convenient up to ∼500,000 molecules because larger data sets tend to be overwhelming.

To adapt TMAP to billion-sized data sets, we now report a “nested TMAP” tool consisting of a primary TMAP displaying similarity relationships between a set of cluster representatives, each of which is linked to a secondary TMAP displaying the corresponding cluster content. In the ideal case, the primary TMAP and secondary TMAPs are of similar size, which allows us to display a database of 10^N^ molecules with TMAPs of approximately 10^N/2^, i.e., 100,000 molecules per TMAP for a database of 10 billion molecules. The primary TMAP representing cluster representatives and the secondary TMAPs displaying cluster contents are displayed in an interactive web-based format supported by the applications Faerun and SmilesDrawer.^22,23^ This setup makes it possible to navigate a data set of billions of molecules interactively from a global overview display in the primary TMAP down to each single molecule within each secondary TMAP (Figure 1).

**Figure 1:**
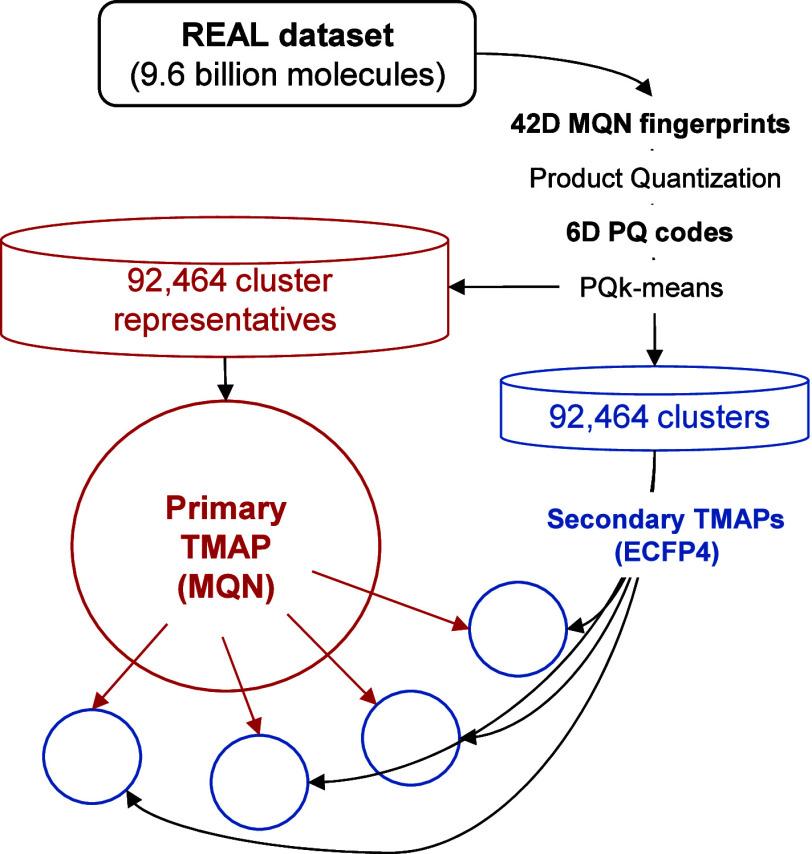
Clustering and nested TMAP layout principle exemplified with the 9.6 billion molecules REAL data set.

A key challenge of our nested TMAP approach to visualize billions of molecules is the initial clustering of the data set. The choice of clustering method is tightly coupled to the nested TMAP design. Because each cluster representative in the primary TMAP is linked to one secondary TMAP, the partition must (i) have a user-specified number of clusters, so that the size of the primary TMAP can be set a priori, and (ii) yield clusters of roughly uniform size with few or no singletons, so that every secondary TMAP contains a meaningful number of molecules. Density- and threshold-based methods such as BitBIRCH^24^ and BitBIRCH-Lean,^25^ recently demonstrated on up to one billion molecules, fit neither constraint: they operate on molecules represented as substructural fingerprints (ECFPs) compared by Tanimoto similarity, so the number of clusters is not set in advance but instead emerges from a user-supplied merge threshold. In the example reported for ∼1 billion ZINC-22 molecules at a 0.5 threshold (after several merging phases), BitBIRCH-Lean produced 2.65 million clusters, only 31% of which contained more than 100 molecules,^25^ which would not be suitable for our nested TMAP approach. BitBIRCH-Lean also reported a peak memory usage of 55 GiB, impractical at 10-billion scale on a standard workstation.

Here, we chose to represent the molecules with MQN (molecular quantum numbers), a 42 dimensional count vector describing atom and bond types, polar groups and topological features, and which we had previously found useful to create maps of large molecular data sets by PCA.^26–29^ To enable processing of a 10 billion size data set, we form clusters using PQk-Means,^30^ which is a k-means-like partition algorithm that operates on a product-quantized Euclidean representation of a higher-dimensional scalar vector representations such as MQN. This approach is computationally efficient for very large data sets including molecular data sets because it avoids the use of computationally expensive pairwise similarity matrices or graph representations.^31–34^

We exemplify our visualization approach with the 9.6 billion molecules in the REAL database made available by Enamine Ltd.,^35^ which is a subset of a larger data set of 76.9 billion products possible by combining a set of building blocks via known reactions, filtering by the drug-likeness criteria Lipinski’s Rule of Five and Veber’s guidelines (MW: ≤500, *S*log *P* ≤ 5, HBA ≤ 10, HBD ≤ 5, Rotatable Bonds ≤ 10, and TPSA ≤ 140).^36,37^ This combinatorial data set is typical of the large libraries currently in use in early phase drug discovery. The nested TMAP example realized here is accessible at https://chelombus.gdb.tools/databases/real-database.

## Methods

The overall workflow consists of four computational stages: (1) MQN calculation from SMILES, (2) product quantization (PQ) codebook training and PQ encoding of all MQNs into PQ codes, (3) clustering of PQ codes by PQk-Means to obtain the cluster assignments and representatives, and (4) visualization by a primary TMAP over cluster representatives with nested secondary TMAPs for the cluster content.

### Molecular Data Preparation

The Enamine REAL Database (9.6 billion structures) was downloaded as SMILES strings from the Enamine Web site in March 2025. To remove ordering effects arising from the default heavy-atom-sorted input, the file was first randomly permuted. Molecular quantum number (MQN) fingerprints^26^ were then computed for every molecule using the RDKit implementation of the 42 MQN indices (RDKit version 2025.09.3) via the rdMolDescriptors module without further preprocessing. These were then used as an input for PQ encoding.

### Product Quantization

PQ was implemented directly in Python following the formulation by Jégou et al.^38^ and the procedure described by Matsui et al.^30^ Each MQN vector $x\in N_{0}^{42}$ was decomposed into *m* = 6 contiguous subvectors of dimension seven. Transforming MQN vectors into PQ codes comprises two operations, a one-time codebook training step and a scalable PQ encoding step.

### Codebook Training

For each of the six subspaces, a separate codebook of *L* = 256 centroids was learned by k-means clustering using k-means++ initialization, Euclidean distance (L2), and 20 iterations. Codebook training was performed on a random subsample of 50 million MQN vectors. We used *m* = 6 subvectors for PQ because MQN fingerprints are 42-dimensional, and the number of subvectors must divide 42 evenly. This gave subvectors of dimension 7, which provided a practical balance between the compression and quantization quality. Lower values of *m* would produce coarser quantization, whereas higher values would increase the code length with limited practical benefit for this descriptor. With *m* = 6 and 256 codewords per subvector, each molecule was represented by a compact 6-byte code while preserving sufficient distance resolution for clustering (Figure 2A).

**Figure 2:**
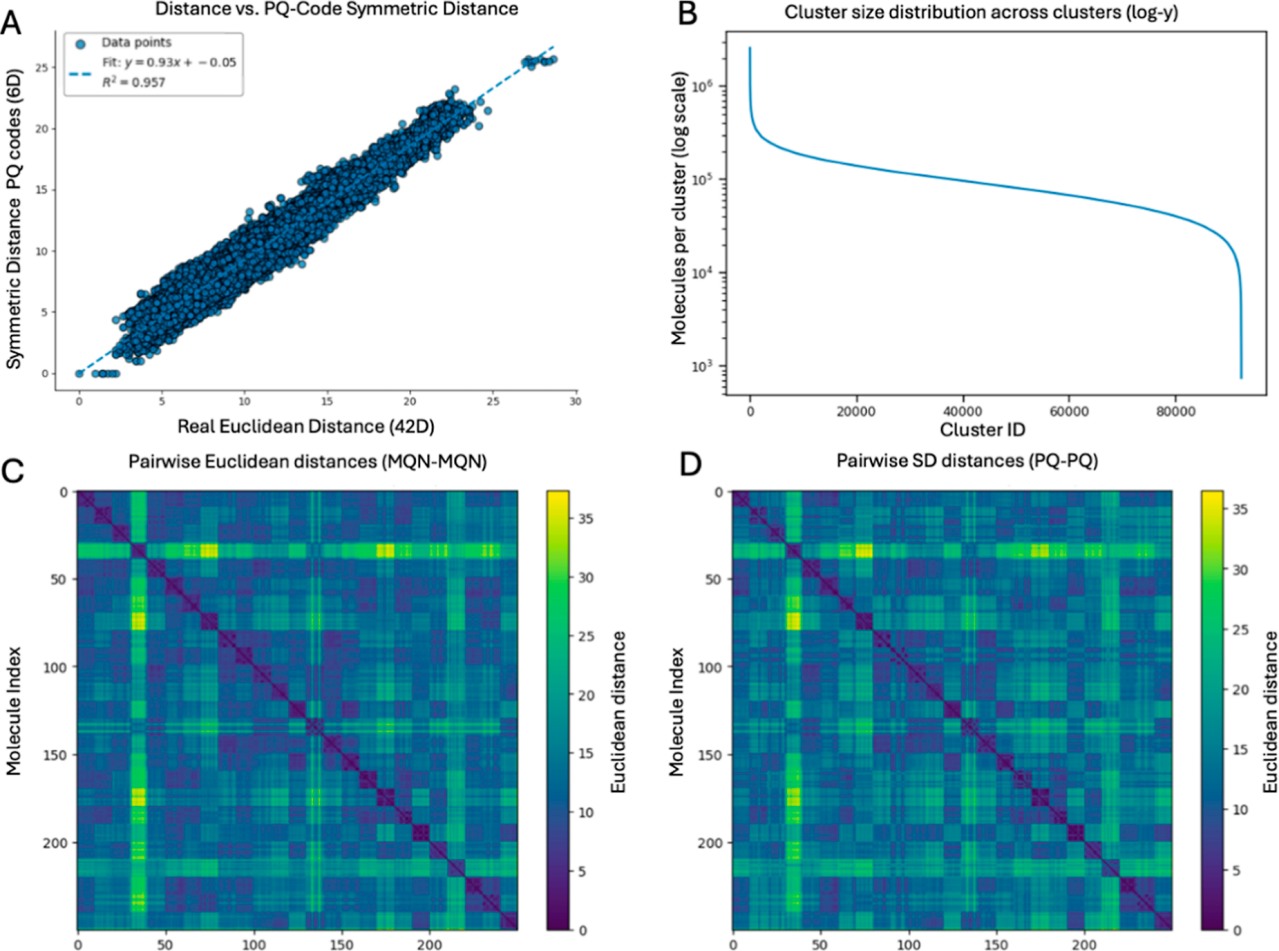
(A) Plot showing the relation between the Real Euclidean distance between a set of MQN vectors (42D) and the symmetric distance (SD) distance between their corresponding PQ codes (6D) to a reference molecule. (B) Cluster size distribution across clusters, sorted by decreasing size. Cluster size: 103,368 ± 76,184 molecules, smallest cluster: 737 molecules, largest cluster: 2,577,108 molecules. (C,D) Heatmap for the pairwise Euclidean Distances between MQN fingerprints and SD (approximate Euclidean) for the PQ Codes for 10 randomly selected molecules from 25 randomly selected clusters. The diagonal squares correspond to within-cluster distances, whereas the rest of squares are between-cluster distances.

### PQ Encoding

After training, all 9.6 billion MQN vectors were reloaded in consecutive shards and encoded into PQ codes. For each molecule, each of the six subvectors was assigned to its nearest centroid by Euclidean distance, yielding a six-integer tuple (*c*_1_,*c*_2_,···,*c*_6_) where each *c*_*i*_ ∈ {0,···,255} indexes a codeword in the corresponding subspace.

All computations were performed on a single workstation (AMD Ryzen 7 8700F, 64 GB RAM, NVIDIA RTX 4070 Ti 16GB). PQ codebook training (50 million MQNs) required approximately 2 min on the GPU (Table 1). Encoding was performed in configurable batches that stream through the GPU, so peak VRAM is bounded by the chosen batch size and is independent of the number of vectors being encoded. In our setup, the GPU kernel sustained ∼6 million molecules/s. End-to-end encoding of the full 9.6 billion data set, including streaming MQN shards from disk, required approximately 45 min (including disk I/O). Peak RAM during encoding is dominated by the shard buffer and is therefore also bounded by a user-configurable parameter.

**Table 1:** Time, Peak VRAM, and Peak RAM Required for PQ Codebook Training (Fit) and PQ Encoding (Transform) for Different Training Subsample Sizes (*N*)­^a^

*N*(10^6^)^b^	fit (s)	transform (s)	peak VRAM (GiB)^c^	peak RAM (GiB)
10	22.56	1.86	6.03	3.5
30	71.96	4.99	6.63	8.74
50	120.73	8.46	7.22	13.95
100	265.04	16.76	8.71	26.56

a The fit column reports the one-time codebook-training cost; the transform column gives the time required to encode the same *N* MQN vectors into *N* PQ codes on GPU.

b MQN fingerprints.

c Codebook training holds the training subsample on the GPU, so its peak VRAM scales with subsample size in the range shown. PQ encoding streams the input in user-configurable batches, so its peak VRAM is bounded by the batch size and independent of the total number of vectors encoded; in practice, this means the encoding step scales to arbitrarily large *N* on any modern GPU.

### Clustering Using PQk-Means

Clustering of all PQ codes into 100,000 clusters was performed using PQk-Means,^30^ a k-means like algorithm that operates directly on PQ codes and uses the SD, a lookup-table based approximation of Euclidean distance. The clustering proceeds in two stages following the recommended PQk-Means workflow. In the first stage, the model was trained on a uniform random subsample of one billion PQ codes to optimize the placement of the 100,000 centroids. Using our GPU implementation, this step required approximately 194 min. The same step on the reference C++ CPU implementation^30^ would have required 7 days and 5 h. In this process, VRAM is set by the batch configuration rather than by any *N*. Peak RAM on the other hand is dominated by holding the PQ-code matrix in memory and scales linearly with the training subsample (13.9 GiB for a 1 billion code subsample used here; Table 2).

**Table 2:** Time and Peak RAM for Centroid Training (Fit) and Cluster Assignment (Predict) for Different PQ-Code Subsamples Sizes *N* and Centroid Counts *K*

*N* (PQ codes)	*K* (num. clusters)	fit (min)	predict (min)	total (min)	RAM (GiB)^a^
100 M	20,000	5.58	0.64	6.22	3.26
100 M	50,000	12.33	1.58	13.91	3.38
100 M	100,000	23.57	3.16	26.73	3.70
250 M	20,000	11.79	1.316	13.11	4.73
250 M	50,000	21.93	3.25	25.18	4.87
250 M	100,000	41.63	6.48	48.12	5.09
500 M	20,000	24.02	2.71	26.74	7.41
500 M	50,000	52.39	6.71	59.10	7.32
500 M	100,000	85.49	13.39	98.87	7.44
1 B	20,000	47.00	5.32	52.32	14.03
1 B	50,000	102.92	13.15	116.07	14.00
1 B	100,000	167.75	26.24	193.99	13.90
2 B	20,000	96.15	10.66	106.81	27.29
2 B	50,000	206.12	26.33	232.44	27.32
2 B	100,000	336.09	52.52	388.62	27.30

a Peak RAM is dominated by holding the PQ-code subsample in memory and therefore scales linearly with *N*. Peak VRAM is not reported in this table because it is set by the user-configured GPU batch size rather than by *N*.

In the second stage, the remaining molecules (encoded as PQ codes) were assigned to the nearest centroids in streaming batches on the GPU. This produced 92,464 populated clusters. Assignment was performed with the same SD metric. The full 9.6 billion assignment required approximately 4.5 h, which results in a 29-fold speedup over our own parallel CPU implementation, which is itself about 2-fold faster than the reference PQk-Means C++ implementation (the latter assigns one cluster at a time in a sequential loop which scales poorly with N). As with PQ encoding, assignment streams through the GPU in user-configurable batches, so peak VRAM is bounded by the batch size rather than by any *N*. For example, a hypothetical single-pass assignment of 2 billion PQ codes would require ∼18.6 GiB of VRAM while running two 1 billion batches instead keeps peak VRAM below 10 GiB at only a small increase in wall time. Peak RAM during assignment was 13.9 GiB. For each cluster, the representative molecule was defined as the element whose PQ code had a minimal SD to the cluster centroid.

### Visualization Using TMAP

A primary TMAP was constructed from the 92,464 representative molecules. For each representative, its original 42-dimensional MQN vector was used as embedding. An approximate k-nearest neighbor graph was computed and used to create the TMAP. Nodes were colored by structural or physicochemical MQN features (e.g., number of rings, heavy atom count, fraction of aromatic atoms). For each cluster, a secondary TMAP was constructed by using the molecules belonging to that cluster. These secondary TMAPs were generated from the ECFP4 fingerprints.

## Results and Discussion

### Clustering of a Billion-Sized Data Set

To cluster the 9.6 billion molecules in the REAL data set, we represented the molecules as 42-dimensional MQN vectors and used PQ to compress these vectors to 6-byte PQ-codes. This method is computationally efficient, produces relatively homogeneous cluster sizes, and can be computed on a single workstation (see Methods for details). As anticipated, the SD between PQ-codes was well correlated with the Euclidean distance between MQN-vectors, showing that PQ preserved distance relationships between molecules while reducing dimensionality by 7-fold (Figure 2A).

Clustering with *k* = 100,000 was performed using the PQk-Means implementation,^30^ operating directly on PQ codes and the associated SD approximation to Euclidean distance, using a subset of one billion molecules (see Methods for details). Trained centroids were then used to assign the rest of the molecules, via their PQ codes, to clusters in the streaming mode. In practice, not all learned centroids were populated after assignment (92,464 of 100,000), which is a standard result of the method. Nevertheless, cluster sizes were well equilibrated overall, with 95% of the clusters in the range of 26,000 to 234,000 molecules (Figure 2B).

Cluster quality was evidenced in pairwise-distance heatmaps, where the within-cluster blocks along the diagonal display systematically lower distances than off-diagonal blocks (Figure 2C,D). In addition, within-cluster similarity was compared to between-cluster similarity by computing distances from random molecule pairs sampled within and across clusters. Analyzing mean within-cluster versus between-cluster distances using Euclidean and Manhattan distances on MQN fingerprints and SD on PQ codes showed that molecules assigned to the same cluster were, on average, substantially closer than molecules drawn from different clusters (Table 3).

**Table 3:** Euclidean, Manhattan, and Symmetric Distance between MQN Fingerprints (Euclidean and Manhattan) or PQ-Codes (SD) from 10 Randomly Selected Molecules from 100 Randomly Selected Clusters

metric	within-cluster (mean ± SD)	between-cluster (mean ± SD)
Euclidean distance (MQN)	4.27 ± 1.63	11.61 ± 3.06
Manhattan distance (MQN)	16.79 ± 8.15	50.14 ± 14.49
Symmetric distance (PQ-codes)	4.68 ± 2.63	12.5 ± 3.54

Cluster quality was further assessed by examining whether molecules grouped within the same PQk-Means cluster exhibited a narrow spread of physicochemical properties and structural descriptors (Table 4). Indeed, this was the case across most descriptors, as indicated by low coefficients of variation (CV), for MW and HAC, which account for the largest variance of the MQN descriptors. Polarity was also largely preserved, with TPSA showing a median CV of 0.11, while FCsp3 displayed a greater heterogeneity. For discrete count descriptors with small typical means (notably HBD and, to a lesser extent, aromatic atoms), CV showed longer upper tails that were largely driven by a division by a small mean. In such cases, the interquartile range (IQR) and range provided a more reliable picture of within-cluster variability. For example, HBD had IQR = 0 at the median despite very large 95th percentile CV, reflecting that most clusters are internally consistent in HBD. Aromatic atom count showed the weakest connection among the reported descriptors, reflecting the fact that aromatic atoms are not counted explicitly in MQN descriptors.

**Table 4:** Cluster Dispersion Statistics for Different Molecular Descriptors

descriptor^a^	coefficient of variation 5th %/median/95th %	interquartile range 5th %/median/95th %	range 5th %/median/95th %
MW	0.03/0.05/0.1	14/24/44	112/159/254
HAC	0.02/0.03/0.06	0/1/2	4/7/11
rings	0.02/0.04/0.06	0/1/2	4/7/11
RBC	0.06/0.12/0.25	0/1/2	2/5/8
HBD	0.08/0.32/7.92	0/0/1	1/3/4
HBA	0.12/0.16/0.28	0/1/2	4/6/7
TPSA	0.08/0.11/0.22	5.1/12.6/18	50/69.5/90.1
FCsp^3^	0.02/0.09/0.31	0/0/1	1/2/3
aromatic atoms	0.17/0.33/5.8	0/4/6	6/12/20

a MW = molecular weight, HAC = heavy atom count, rings = ring count, RBC = rotatable bond count, HBD = H-bond donor atom count, HBA = H-bond acceptor atom count, TPSA = topological polar surface area (Å), FCsp^3^ = fraction of sp^3^ carbon atoms. Dispersion was quantified by using three complementary statistics: range (sensitive to outliers), interquartile range (IQR, robust spread of central 50%), and coefficient of variation (CV = σ/μ relative spread).

### Nested TMAP Visualization

The nested TMAP exploits the MQN and ECFP4 complementarity. The primary TMAP is laid out by MQN similarity between cluster representatives, reflecting whole-molecule composition trends that are well captured by a 42-dimensional integer count vector. Each secondary TMAP is laid out by ECFP4 similarity within a single cluster, surfacing substructural relationships at a resolution that a compact count fingerprint cannot provide. MQN and ECFP4 therefore play distinct but complementary roles rather than competing representations. The primary TMAP was generated from the 92,464 cluster representatives, using MQN-fingerprints as molecular representation. Its computation was fast compared to clustering and required on the order of 1 min. Color-coding the primary TMAP by representative properties revealed coherent subregions associated with MQN-derived structural trends, such as ring count, aromaticity, and size-related descriptors. Each node of the primary TMAP was linked to a secondary TMAP representing the corresponding cluster contents. Secondary TMAPs were generated for cluster contents using ECFP4 fingerprints to provide a more detailed insight into each cluster (Figure 3). Generating a single secondary TMAP required approximately 1 min or less. Precomputing secondary TMAPs for all clusters therefore represents a substantial computational effort on a single workstation. In practice, secondary TMAPs might be computed on demand for clusters of interest or generated in parallel on a computing cluster, depending on whether the goal is interactive web deployment or offline analysis. Samples of cluster contents are illustrated in Figure 4.

**Figure 3:**
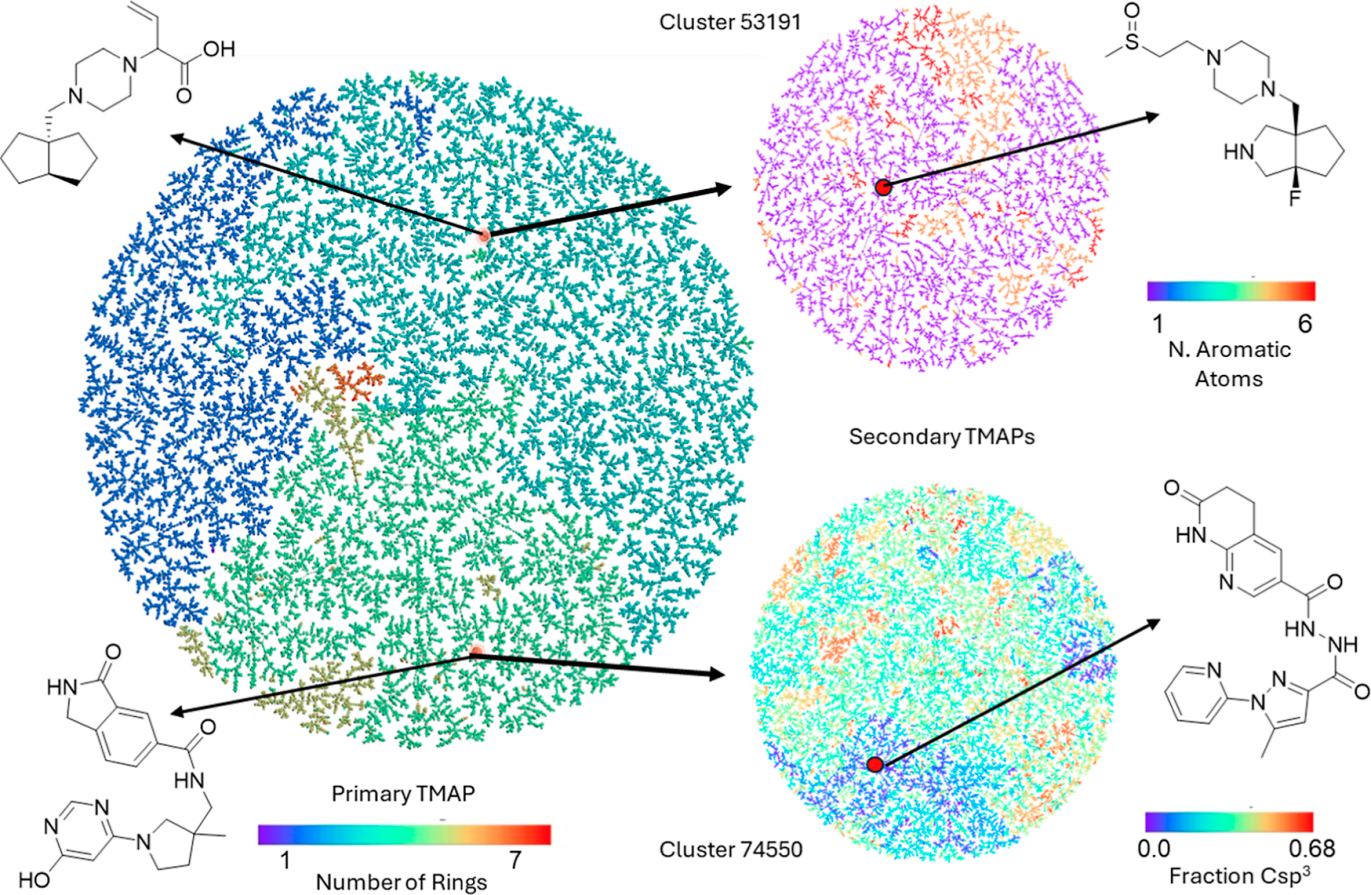
Illustration of the nested TMAP visualization of the REAL 9.6 B data set. The primary TMAP at left is organized by MQN similarity and features 92,464 cluster representatives. Each cluster representative links to a secondary TMAP featuring the cluster contents organized by ECFP4 similarity. Two example clusters are shown with the cluster representative at left and one randomly picked molecule from each cluster TMAP at right. The interactive nested TMAP is accessible at https://chelombus.gdb.tools/databases/real-database.

**Figure 4:**
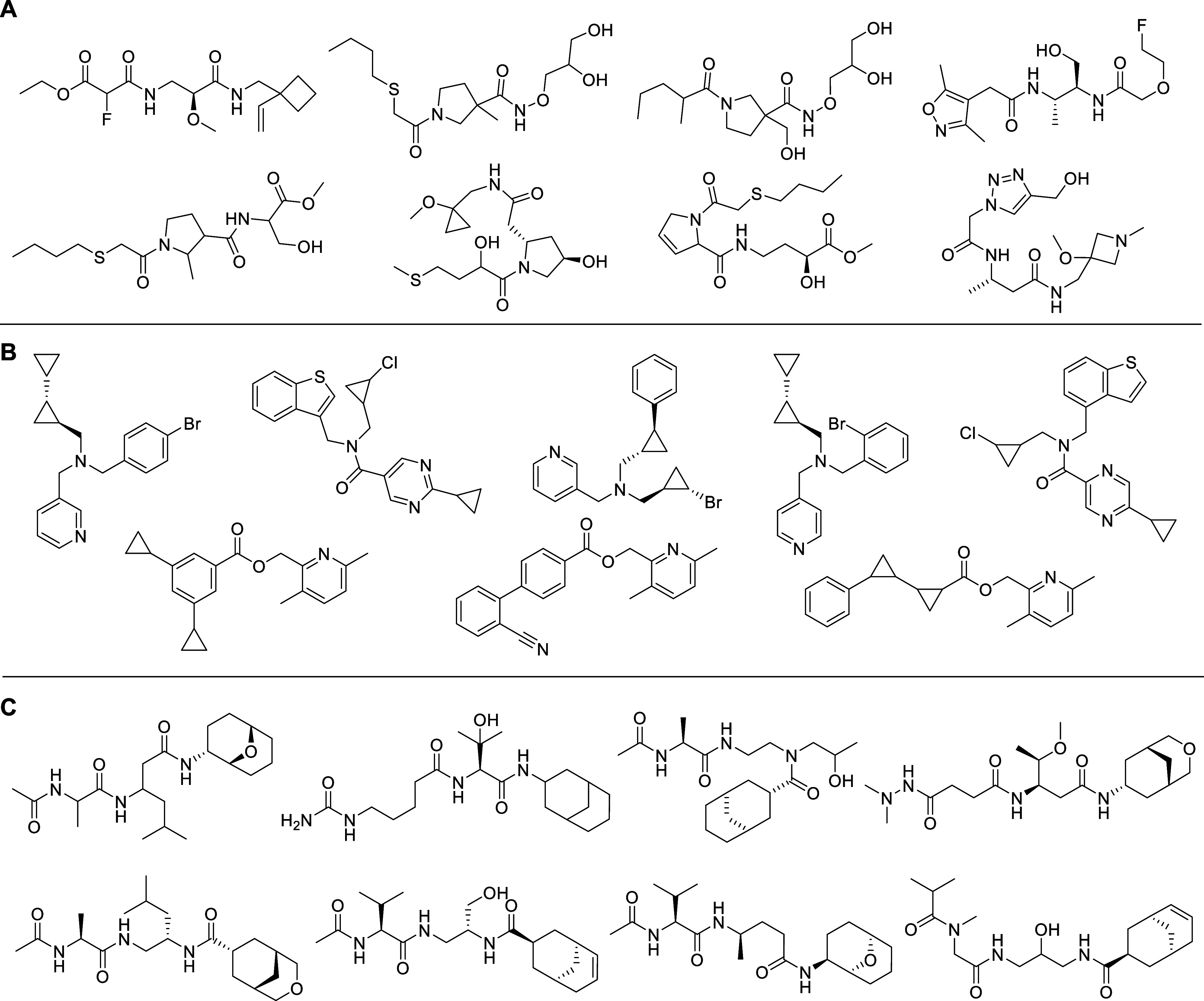
Samples of molecules from cluster contents. (A) Cluster ID 9547 containing 2,577,108 molecules. (B) Cluster ID 84267 containing 84,801 molecules. (C) Cluster ID 76115 containing 737 molecules.

## Conclusion

The nested TMAP framework described above enables an interactive exploration of billion-sized chemical data sets on commodity hardware. The method combines an interpretable, low-dimensional count fingerprint (MQN) with PQ and PQk-Means clustering to organize an ultra large chemical space into a set of cluster representatives. Visualization is achieved by a primary TMAP describing relationships between cluster representatives, where each node links to a nested secondary TMAP displaying the corresponding cluster content. The resulting representation provides a global overview of chemical diversity while preserving access to individual molecular structures, as exemplified with the 9.6 billion REAL data set. Note that adding a new batch of molecules to an existing nested TMAP visualization of a data set would not require recomputing the cluster centers and the primary TMAP if the underlying distribution of the batch remains similar to that of the entire data set, so that only part of the secondary TMAPs would need to be recomputed.

## Data Availability

All code written for PQ implementation, TMAP generation, and preprocessing is available at https://github.com/afloresep/Chelombus. Trained models and tutorials are also provided there. The data sets used to train the models can be downloaded from https://enamine.net/compound-collections/real-compounds/real-database.
